# The Application of Graph Theoretical Analysis to Complex Networks in Medical Malpractice in China: Qualitative Study

**DOI:** 10.2196/35709

**Published:** 2022-11-03

**Authors:** Shengjie Dong, Chenshu Shi, Wu Zeng, Zhiying Jia, Minye Dong, Yuyin Xiao, Guohong Li

**Affiliations:** 1 School of Public Health Shanghai Jiao Tong University Shanghai China; 2 Shuguang Hospital Affiliated to Shanghai University of Traditional Chinese Medicine Shanghai China; 3 Center for Health Technology Assessment China Hospital Development Institute Shanghai Jiao Tong University Shanghai China; 4 Department of International Health Georgetown University Washington, DC United States; 5 Renji Hospital School of Medicine Shanghai Jiao Tong University Shanghai China; 6 China Hospital Development Institute Shanghai Jiao Tong University Shanghai China

**Keywords:** medical malpractice, complex network, scale-free network, hub nodes, patient safety management, health systems

## Abstract

**Background:**

Studies have shown that hospitals or physicians with multiple malpractice claims are more likely to be involved in new claims. This finding indicates that medical malpractice may be clustered by institutions.

**Objective:**

We aimed to identify the underlying mechanisms of medical malpractice that, in the long term, may contribute to developing interventions to reduce future claims and patient harm.

**Methods:**

This study extracted the semantic network in 6610 medical litigation records (unstructured data) obtained from a public judicial database in China. They represented the most serious cases of malpractice in the country. The medical malpractice network of China was presented as a knowledge graph based on the complex network theory; it uses the International Classification of Patient Safety from the World Health Organization as a reference.

**Results:**

We found that the medical malpractice network of China was a scale-free network—the occurrence of medical malpractice in litigation cases was not random, but traceable. The results of the hub nodes revealed that orthopedics, obstetrics and gynecology, and the emergency department were the 3 most frequent specialties that incurred malpractice; inadequate informed consent work constituted the most errors. Nontechnical errors (eg, inadequate informed consent) showed a higher centrality than technical errors.

**Conclusions:**

Hospitals and medical boards could apply our approach to detect hub nodes that are likely to benefit from interventions; doing so could effectively control medical risks.

## Introduction

### Background

Medical malpractice is a complex issue involving many different elements and their mutual relationships. The interacting elements in medical malpractice could comprise individuals (such as physicians and patients) and institutions (such as hospitals). These elements play particular roles in medical malpractice and have strong or weak connections with it. For example, physicians with poor malpractice records are more likely to stop practicing medicine, switch to smaller practice settings [1,2], or practice defensive medicine. Most malpractice cases are brought against the same physician and occur in the same specialty [3-5]. Owing to the complexity of the topic, it is difficult to describe the organizational themes in medical malpractice using a model or mathematical formula.

The construction and structure of networks may help to understand the complex issues—network thinking focuses on relationships among entities rather than on the entities themselves. Network thinking provides novel ways to address difficult problems such as how to control epidemics; how to target diseases that affect complex networks in the body; and, more generally, what kind of resilience and vulnerabilities are intrinsic to natural, social, and technological networks as well as how to exploit and protect such systems [6]. Similarly, establishing a reasonable medical malpractice network is of great significance for examining common patterns among entities. For example, AIDS network studies [7-9] have suggested that safe sex campaigns, vaccinations, and other interventions should be mainly targeted at hubs in sex contact networks. With complex networks and limited resources, hub targeting would be the most cost-effective strategy [10,11].

Medical malpractice in China is an issue that needs immediate attention. According to statistics from the Supreme Court of China [12], there are >10,000 medical lawsuits each year, and the number of cases has increased markedly. The impact of medical litigation cases is excessive. Wang et al [13] and Li et al [14] estimated that approximately 70% of medical lawsuits in China were related to alleged inadequacies in the quality of health care. However, in Denmark and Sweden, medical litigation cases resulting from insufficient quality of care accounted for only approximately 50% of medical lawsuits [15,16]. The frequent occurrence of such cases will not ease the current tense physician-patient relationship [17,18] and could induce defensive medical behavior. It is believed that defensive medicine either promotes the rise of medical costs or reduces care quality. Unlike the soaring insurance costs caused by the “malpractice crisis” in Europe and the United States, the cost to China’s insurance system appears to be stable, but there may be a huge impending crisis. In China, health care services are mainly provided by public hospitals. Hospitals generally do not purchase commercial insurance and, thus, they bear the medical risks. The lack of a medical risk-sharing mechanism makes it more likely that payments incurred by lawsuits will be potentially diverted from patients’ medical costs; this will make the direct and indirect costs of malpractice more difficult to control.

Studies on medical malpractice have mostly investigated what motivates patients to sue and how malpractice claims affect physicians’ behavior—the aim has been to determine the incentives to practice defensive medicine and change treatment patterns. However, the analytic methods of such studies have been limited to describing characteristics, time trends, and associations; each method has had potential drawbacks and limitations. The complex network theory can provide methodological support for understanding the complexity of a health care system; however, few studies have focused on interactive behavior in medical malpractice in terms of network thinking.

### Background Literature

In 2000, the US Institute of Medicine released a report titled “To Err Is Human: Building a Safer Health System” [19], which attracted public attention to incidents of medical malpractice. In recent years, in the United States and Europe, there has been an increase in the number of malpractice claims against health care providers as well as in the amount of payment awarded to plaintiffs. Many descriptive studies have undertaken retrospective analyses of claims [20] with respect to specialties [4,19,21], regional factors [13,22], and medical errors [14,19,23]. On this basis, correlation studies have been conducted, including the following areas: correlations between physician traits and claims [24-26], quality of care and claims [27,28], and medical insurance costs and the medical liability system [29,30]. In the United States in particular, researchers have attempted to explain the sudden increases in claims and sharp rise in insurance premium rates; some believe that such trends may have been caused by a decline in care quality or a lack of efficient incentive schemes provided by legislation. Many studies on medical malpractice have examined the characteristics of liability systems and their ability to prevent negligence and make policy recommendations for ongoing system reform. Other studies have focused on analyzing the impact of medical malpractice on physicians’ behavior and their motives for defensive medicine [31-33].

Health care is complex. Renkema et al [33] identified the complexity of care as a major factor affecting the relationship between malpractice claim risk and physicians’ behavior. Given the complexity of health care, complex theory has been applied to studies on health in many ways. A much-cited article in *The British Medical Journal* by Plsek and Greenhalgh [34] has provided a powerful impetus for the application of complex theory in the field of health. This introductory article argued that, to cope with the growing complexity of health care, linear models had to be abandoned and unpredictability accepted, calling for consideration of the complexity of health services. As an emerging field in complexity research, complex network theory abstracts complex systems into networks, with nodes and connected edges to analyze topology and common patterns for systems. Two well-studied models in complex network theory are the small-world network and scale-free network models [10,35,36]. Originally described in social networks, the small-world property means that the distance between any 2 nodes in a network is unexpectedly small. The scale-free network property means that numerous weakly connected nodes (noninfluential nodes) coexist with a few highly connected nodes (influential nodes).

The complex network theory has been used for studies in evaluating health policy, the spread of infectious diseases, and the mechanism of physiological systems. Yue et al [37] investigated the implementation process of essential drug policy in 3 rural areas in China through the lens of complexity. The authors identified the importance of adaptiveness and self-organizing behavior as well as the role of nonlinear feedback loops in the implementation process. In 2001, a research team of sociologists and physicists from Sweden found that the network of human sexual contacts showed a scale-free structure [7]. Other research has drawn similar conclusions. These findings have provided valuable information for epidemic control, such as with AIDS—in the case of limited resources, it is most cost-effective to prioritize behavioral education or vaccination of the hub node (the most influential node) in the sex network. Several studies in brain science have found that human and other animals’ brain structures and functional networks have the following features: small-world topologies [38-41], highly connected hub nodes [42], and modular partitions [43]. There has been limited research on applying complex network theory to medical malpractice. This study used data on medical litigation from China and applied the complex network theory aiming to construct the topology of a medical malpractice network.

## Methods

### Overview

In this study, we constructed a knowledge graph (KG) to represent the medical malpractice network of China (MMNC). Our null hypothesis was that claims are random events—attributable to bad luck with random frequency. Correspondingly, our alternative hypothesis was that medical malpractice is not random; this reflects the belief that hospitals or physicians with multiple malpractice claims are more likely to be involved in new claims. As medical malpractice is a complex issue, this study applied the complex network theory, which provided the methodological support for understanding interactive behavior in medical malpractice. Specifically, this study extracted the semantic network in 6610 medical litigation records (unstructured data) obtained from a public judicial database in China. They represented the most serious cases of malpractice in the country. The MMNC was presented as a KG; it uses the International Classification of Patient Safety from the World Health Organization (WHO-ICPS) as a reference.

### Construction of the Malpractice Network

#### Overview

**Figure 1:**
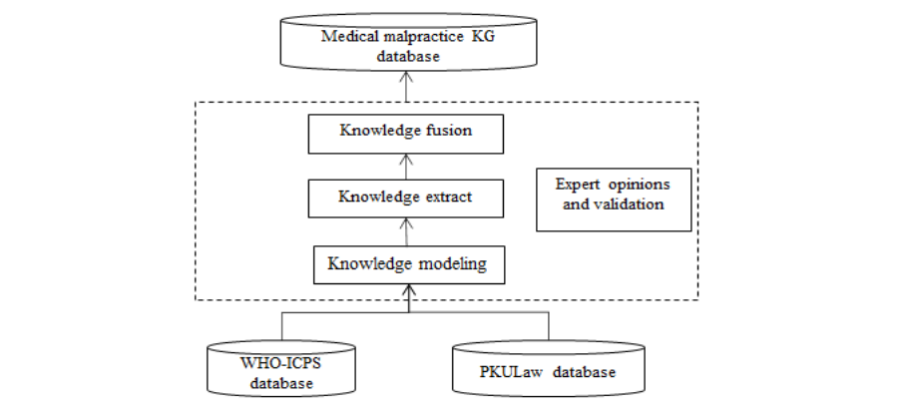
Process of constructing the medical malpractice knowledge graph (KG). The International Classification of Patient Safety from the World Health Organization (WHO-ICPS) is a conceptual framework with an ontological basis. However, the WHO-ICPS was not a complete classification at that time. We adopted and localized several key concepts from the WHO-ICPS (details in Table 1).

A complex network can be represented as a KG, which is widely used to express a semantic network. A difficulty in this regard is how to generate an effective, reliable KG. This study followed the general steps of KG development shown in Figure 1. In that process, this research adopted top-down logic (ie, designing the data model first; filling the specific data to the model; and, finally, forming a KG). We stored the KG in Neo4j Community Edition (version 3.5.5; Neo4j, Inc) [44], which is the world’s leading graph database and has been widely used because of its higher performance. The structural medical malpractice network can be represented as a KG through the following 4 steps.

#### Step 1: Knowledge Modeling

The knowledge model is the top-level design with a KG—it determines the range of data collected and the structure of the data. From a technological perspective, it defines the *schema* of the KG. In this study, we examined dynamic development in the MMNC—we attempted to determine the underlying mechanism, and the logic can be summarized in chronological order as follows. Patients seek medical advice because of illness. In the case of several medical errors or relatively unsatisfactory outcomes, patients become discontented with the efforts of medical providers and have the incentive to undertake legal action. Each malpractice claim concludes with a legal judgment. The patient, medical provider, and court were considered as stakeholders in the MMNC.

To extract medical litigation texts from the database in China, we referred to the WHO-ICPS [41,45], which offers a conceptual framework using an ontological basis. All definitions and the knowledge model were clarified after repeated discussions by an expert panel (details are described in step 4). The WHO-ICPS is an internationally standardized domain ontology, and it can be directly used as a model when constructing a KG for patient safety. Therefore, this study examined the WHO-ICPS to help construct a theoretical model in step 1.

The actual practice knowledge modeling adhered to the following steps.

First, we defined the network nodes and their properties. The aforementioned stakeholders were classified and served as the nodes. Furthermore, nodes were assigned several properties that were used to form a comprehensive description of the nodes.

Second, we estimated a continuous measure of the association among the nodes. Given that medical litigation cases were in text format, specific sentences that described the relationships among key concepts were abstracted as the relationships. For example, we abstracted “seek medical service” as a relationship from the sentence “Patient A sought medical service from the oncology department at Hospital B.”

Third, we generated an association matrix by compiling all pairwise associations among nodes. We kept the relationships directed (in chronological order) to allow us centrality analysis and weighted (weight was the number of relationships).

#### Step 2: Knowledge Extraction

From step 2, we obtained information about the nodes and their relationships and properties. Records relating to medical lawsuits were in the form of unstructured data; they covered the contents of patients’ medical records, medical expert opinions, and court decisions. To extract knowledge from the unstructured litigation data, we used the knowledge model built in step 1 as our structural ontology. Through manual questionnaire entry and crawler codes, we structuralized all the litigation data.

#### Step 3: Knowledge Fusion

This step solved the problem of inconsistent data quality and structure. We adopted a top-down KG construction method. We used a single data source to avoid, to some extent, such problems as uneven information quality and lack of a hierarchical structure. However, during step 2 (especially with manual data entry), there were differences in understanding among data entry operators. To address these problems, we conducted group training before data entry and answered any questions promptly during the process of data entry. After completing the entry, we undertook data verification to ensure reliability; 20% (1322/6610) of the records were double entered (details are provided in the Graph Theoretical Approaches to Network Analysis section).

#### Parallel Step: Expert Opinions and Validation

Expert judgment techniques are useful for various reasons, including cost and lack of sufficient observations for quantification with real observed data. We sought expert opinion with the aforementioned 3 steps—especially where little or no data were available for a node or relationship of interest or the existing data were unreliable.

We selected the experts based on their recognized proficiency and experience in medical malpractice, patient safety, KGs, and IT related to this study. We chose our panel of experts from a number of reputable Chinese medical institutions, including the China Hospital Development Institution of Shanghai Jiao Tong University and the School of Public Health of Shanghai Jiao Tong University. All the experts had access to the medical litigation data stored in the PKULaw database and were involved in all stages of modeling, extraction, and fusion.

### Data Collection and Preparation

After finalizing the structure of the KG (knowledge model and its graphical representation), we used the available data to quantify the KG. We used the PKULaw database (a publicly available database) as the basis for our study. The database is a national repository of all medical malpractice litigation cases against hospitals and has been admitted by the Supreme Court of China since 2003. As of December 30, 2019, the database covered >76 million litigation cases. All the medical malpractice litigation cases in the database were in text format; however, they all had similar content and structure. Specifically, each case was required to have recorded all the following information: the plaintiff and defendant, any medication involved, any hospital-acquired injury, adverse outcomes, evidence of potential negligence, legal questions, and relevant legislation and judgment.

**Figure 2:**
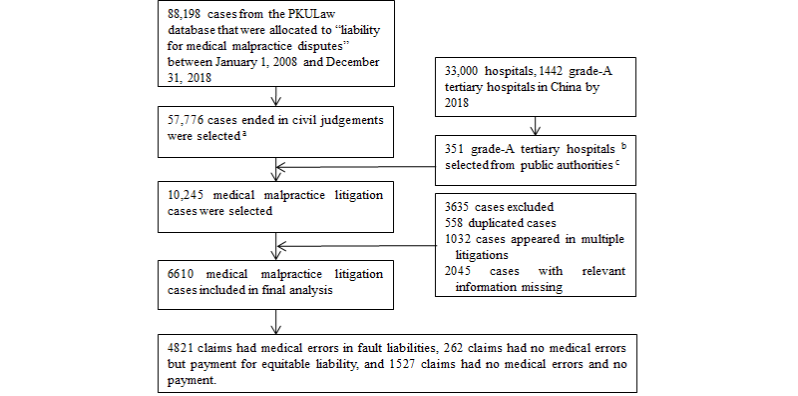
Flowchart of selection of medical malpractice claims in China from 2008 to 2018. a: Civil ligations have three results in China: civil ruling, civil judgment, and civil mediation in court. Cases that end in a civil ruling or mediation do not record relevant information in detail, especially medical information; thus, we excluded such cases. b: Grade-A tertiary hospitals are the highest-level institutions in China. Our selected 351 grade-A tertiary institutions amounted to only 1.1% of all hospitals in China; however, their total number of admissions in 2018 was estimated to be 28 million. We gathered information mainly from the hospitals’ official websites. These 28 million admissions accounted for 11% of the nation’s total number of admissions in 2018 (254 million, gathered from the China Health Statistics Yearbook [National Health Commission of the People’s Republic of China 2019]). c: Eligibility required that a hospital be on a list of public authorities in any previous year. We included four influential ranking lists by public authorities in China: the Best Hospital Ranking by the Hospital Management Institute of Fudan University, the Science and Technology Evaluation Metrics of Hospitals by the Chinese Academy of Medical Sciences, the Hospital Competitiveness Ranking by the Alibi Hospital Management Research Center, and the Best Clinical Specialty Assessment Ranking by Peking University.

We searched the PKULaw database and downloaded files on litigation cases that were concluded from January 1, 2008, to December 31, 2018, in the category of “liability for medical malpractice disputes.” The inclusion criteria were (1) cases concluded with a civil judgment and related to grade-A tertiary hospitals and (2) tertiary hospitals on one of the ranking lists published by the Chinese public authorities. We filtered the records using each eligible hospital’s name as a keyword. We excluded records where basic information was missing or duplicate records of individual cases. If a case was reported in multiple records, we kept only the record of the final judgment (Figure 2).

### Graph Theoretical Approaches to Network Analysis

#### Overview

To investigate networks systematically, we had to define precisely what we meant by “network.” In the simplest terms, a network is a collection of *nodes* connected by *relationships*. Nodes correspond to the entities in a network and links to the connections among them [46]. If a network has a large number of nodes with complex relationships, it can be called a *complex network*. In network science, the number of relationships coming into (or out of) a node is called the *degree* of that node—that is the most fundamental network measure; most other measures are ultimately linked to node degree [46].

We examined the network structure to gain greater insight into what we were dealing with. Two types of models are often examined: *random* and *scale*-*free* networks. Random networks assume that all connections are equally probable, resulting in a Poisson or bell-shaped degree distribution [47]. A scale-free network assumes that the degree distribution follows a power law [35]. In this study, we plotted the *degree distribution* [36] of the MMNC to gain a preliminary understanding of its architecture. We then conducted a scale-free network test, which allowed us to determine the best-fitting power-law model, test its statistical plausibility, and compare it with alternative distributions using a likelihood ratio test [48]. We analyzed the data using R code posted on the web by Clauset et al [48].

We further examined the topological properties of complex systems, such as centrality [49] and distribution of network hubs [50]. The term “hubs” refers to nodes with high degree or high centrality; the removal of hubs can offer advantages with respect to the MMNC. The centrality metrics used in this study included in-degree, closeness, betweenness, and PageRank; they represented a node’s distance advantage through its direct connection to others, a node being accessible to others, a node being an intermediary between others, and a node’s importance, respectively. In this study, we used the centrality algorithms provided in Neo4j.

#### Degree Centrality

Degree centrality measures the number of incoming and outgoing relationships of a node and, thus, can help us find popular nodes in a network [35]. The degree centrality of a node *i* reflects its connectivity in the network and is written as D(i)=*d_i_*/(N−1), where *N* is defined as the number of nodes and *d_i_* is defined as the degree of node *i*, that is, the number of incoming and outgoing relationships of node *i*.

#### Closeness Centrality

Closeness centrality is a way of detecting nodes that are able to spread information very efficiently through a given network. Nodes with a high closeness score have the shortest distances to all other nodes [51], meaning that they are convenient to reach other nodes. The closeness of node *i* is defined as C(i)=(N−1)/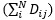 , where N is defined as the number of nodes and *D_ij_* is defined as the shortest path between nodes *i* and *j*. When no path exists between nodes *i* and *j*, *D_ij_* is equal to 0.

#### Betweenness Centrality

Betweenness centrality is a way of detecting the amount of influence a node has on the flow of information in a network, first described by Anthonisse [52] and Freeman [53]. It is often used to find nodes that serve as a bridge from one part of a network to another. For example, people with high betweenness centrality tend to be brokers on social networks by combining different perspectives, transferring ideas between groups. The betweenness of a node *i* reflects its transitivity and is defined as B(i)=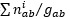, where *g_ab_* is the sum of all the shortest paths between nodes *a* and *b*, 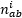 is the number of the shortest paths that pass through node *i*, and *a*≠*b*≠*i*.

#### PageRank Centrality

PageRank centrality measures the transitive influence or connectivity of nodes, and it is used to rank websites in Google search results. For example, the home page usually has the highest PageRank centrality as it has incoming links from all other pages. The PageRank score of node *i* counts the number and quality of links to a page, which determines an estimation of how important the page is and is written as PR(i)=(1–*d*)+*d* (PR[T1]/C[T1]+...+PR[Tn]/C[Tn]), where we assume that a page *i* has pages T1 to Tn that point to it and *d* is a damping factor that can be set between 0 and 1. It was set to 0.85 in this study. C(i) is defined as the number of links going out of page *i*.

### Ethics Approval

The data used in this study were publicly available and considered “not regulated” by the institutional review boards of the relevant hospitals.

## Results

### Conceptual Structure of the KG

**Figure 3:**
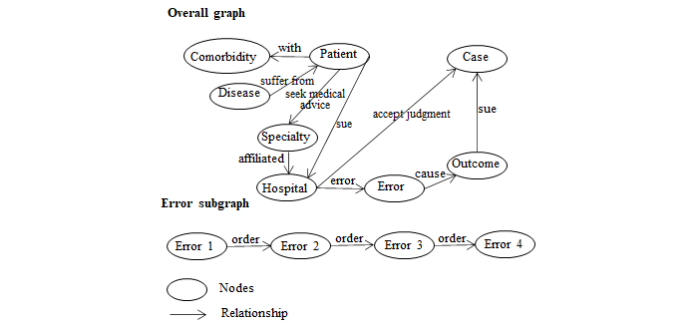
Conceptual knowledge graph representation of the medical malpractice network of China.

We abstracted and integrated 8 key concepts and 9 types of relationships into the conceptual graph representation of the MMNC (the overall graph in Figure 3). Multiple medical errors in a case were connected sequentially by the order of occurrence (error subgraph in Figure 3). For instance, patient A had breast cancer, and she also had diabetes. She sought medical services from the oncology department at hospital B. Owing to a delay in treatment and other risk factors, patient A unfortunately died. A malpractice claim was filed, and hospital B paid compensation according to the legal judgment. All the key concepts in the MMNC are defined in Table 1.

The distribution of the number of relationships per node was highly skewed, with a median of 1 relationship per node. The top 0.78% (149/19,099) nodes accounted for most (28,850/57,700, 50%) relationships in the graph. In the graph, 34.45% (6580/19,099) of nodes had only a single relationship.

**Table 1:** Definitions of nodes and relationships.

Name	Type	Definition	Number
P^a^	Node	Plaintiffs, claims of negligence in the medical service they received This type of node recorded selected attributes of a patient such as patient demographics.	6582
H^b^	Node	Defendants offering medical services for plaintiffs This type of node recorded selected attributes of a hospital such as hospital level or geographic location.	351
S^c^	Node	Physicians’ specialty This type of node recorded selected attributes of a specialty such as type.	38
O^d^	Node	The impact on a patient, which is wholly or partially attributable to an error or a series of errors This type of node recorded the degree of an outcome, which was adapted from Patient Outcome in the WHO-ICPS^e^, including the following^f^: None: patient outcome is not symptomatic, or no symptoms are detected and no treatment is required. Minor injury: patient outcome is symptomatic, symptoms are mild, loss of function or harm is minimal or intermediate but short term, and no or minimal intervention is required. Severe injury: patient outcome is symptomatic, requiring life-saving intervention or major surgical or medical intervention, shortening life expectancy, or causing major permanent or long-term harm or loss of function. Death: on balance of probabilities, death was caused or brought forward in the short term by the error(s). Mental injury only: patient outcome is only mentally symptomatic, and no other symptoms are detected.	5
C^g^	Node	Malpractice claims because of professional misconduct or error or demonstration of an unreasonable lack of skill with the result of injury, loss, or damage to the patient This type of node recorded selected attributes of a claim such as case details or the court.	6610
CD^h^	Node	Comorbidities according to the CCI^i^ This type of node recorded scores on the CCI.	20
E^j^	Node	A failure to carry out a planned action as intended or application of an incorrect plan This type of node recorded types of errors, which was adapted from incident type in the WHO-ICPS and revised by expert opinions, generally classified into “technical error” (related to diagnosis or drugs used) and “nontechnical error” (related to medical records, informed consent, or privacy). More details are provided in Multimedia Appendix 1.	125
D^k^	Node	Disease groups; diseases were classified into 23 categories according to the ICD-10^l^ used by the WHO^m^. This type of node recorded selected attributes of a disease such as its status and group.	5368
With	Relationship	Patients’ comorbidities; links between P and CD	2097
Suffer from	Relationship	Patients’ disease groups; links between P and D	6610
Seek medical advice	Relationship	Patients’ admission specialties; links between P and S	6610
Affiliated	Relationship	The subordinate relationship between admission specialties and hospitals; links between H and S	6610
Error	Relationship	The occurrence of medical errors based on court judgments; links between H and E	4821
Accept judgment	Relationship	Court decision of malpractice claims; links between H and C	6610
Cause	Relationship	Hospitals’ negligence causes patients’ bad outcome; links between O and C	4821
Sue	Relationship	Patients (with bad outcome) bring hospitals to court; links between O and C or P and H	13,320
Order	Relationship	The occurrence order of errors; links between E and E	6201

^a^P: patient.

^b^H: hospital.

^c^S: specialty.

^d^O: outcome.

^e^WHO-ICPS: International Classification of Patient Safety from the World Health Organization.

^f^In practice, we measured “Outcome” by combining the 10 types of relationships based on disability levels, which were classified by the Medical Accident Grading Standard in China (for Trial Implementation since 2002), into four categories: minor injury (injury below the disability level of 5), serious injury (disability level of 1 to 5), death, and mental injury only. The more serious the injury, the lower the disability level; disability level 1 is the most serious injury excepting death.

^g^C: case.

^h^CD: comorbidity.

^i^CCI: Charlson Comorbidity Index.

^j^E: error.

^k^D: disease.

^l^ICD-10: International Statistical Classification of Diseases and Related Health Problems, 10th Revision.

^m^WHO: World Health Organization.

### Distribution of the Malpractice Network

**Figure 4:**
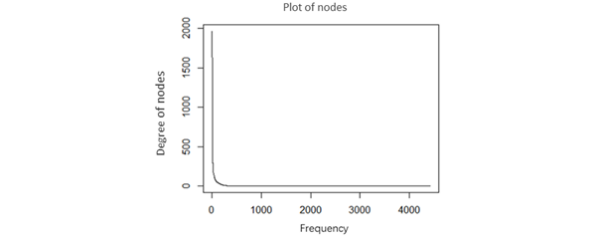
Degree distribution of the network.

In medical malpractice, random events do not occur. The steep curve in Figure 4 shows that the network had many nodes with only a small number of relationships; a few hubs exhibited an extraordinarily large number of relationships. The distinguishing feature of a power law is that there are many small events, and numerous tiny events coexist with a few very large ones. These extraordinarily large events simply do not exist in a bell curve.

In accordance with the method by Clauset [48], we obtained our best-fitting power-law distribution model with the parameters *X_min_*=137 and α=2.463458. After we performed 2500 Kolmogorov-Smirnov tests, 2489 (99.56%) failed to reject the scale-free hypothesis. We also fitted an exponential and log-normal distribution to medical malpractice data and performed a goodness-of-fit test to see if these fits were any good. We obtained our best-fitting exponential distribution model with the parameter λ=0.1889905 and our best-fitting log-normal distribution model with the parameters µ=0.5699136 and σ=1.846312. After we performed 2500 Kolmogorov-Smirnov tests for each distribution model, the results were similar; that is, 100% (2500/2500) rejected the scale-free hypothesis. We concluded that the power-law distribution displayed a good fit to the degree distribution of nodes from the MMNC (ie, it was a scale-free network).

### Hub Nodes in the Malpractice Network

Scale-free networks are characterized by high clustering and skewed degree distributions. Such features predict that each scale-free network will have several large hubs that will fundamentally define the network’s topology (Tables 2 and 3). More information about the sample characteristics is provided in Multimedia Appendix 2.

Table 2 reports the top 10 nodes by degree, closeness, betweenness, and PageRank. Orthopedics, obstetrics and gynecology, emergency medicine, gastroenterology, general surgery, and cancer were ranked as the top specialties in all 4 metrics. On the basis of degree, betweenness, and PageRank, the 3 outcome nodes for death, minor injury, and severe injury were ranked close to the forefront. Specific medical errors appear a number of times in Table 2: inadequate informed consent, delay in treatment, and failure to recognize complications. In general, the results of the 4 centrality metrics were relatively consistent; the nodes that were ranked at the top had a higher degree of coincidence.

Similarly, Table 3 indicates that a few nontechnical errors such as inadequate informed consent and illegible medical records appeared as the top errors with almost all metrics. However, in terms of betweenness, technical errors (including delay in treatment and failure to recognize complications) had higher values. All the top 10 errors with the PageRank metric were nontechnical. In general, the error nodes that were ranked high were relatively consistent, and nontechnical errors were more central than technical errors.

**Table 2:** Top 10 nodes by degree, closeness, betweenness, and PageRank in the overall graph.^a^

Rank	Degree	Closeness	Betweenness	PageRank
1	Death	Orthopedics	Death	Death
2	Minor injury	Emergency medicine	Orthopedics	Minor injury
3	Severe injury	Failure to perform preoperative evaluation^b^	Minor injury	Severe injury
4	Orthopedics	Missed diagnosis^b^	Emergency medicine	Orthopedics
5	Inadequate informed consent^b^	Obstetrics and gynecology	Obstetrics and gynecology	Inadequate informed consent^b^
6	Obstetrics and gynecology	Delay in diagnosis^b^	Gastroenterology	Obstetrics and gynecology
7	Emergency medicine	Gastroenterology	Inadequate informed consent^b^	Delay in treatment^b^
8	Other comorbidities	Inadequate informed consent^b^	Severe injury	Emergency medicine
9	Gastroenterology	Failure to recognize complications^b^	Cancer	Gastroenterology
10	Delay in treatment^b^	Cancer	General surgery	Failure to recognize complications^b^

^a^The definitions of all the nodes can be found in Table 1.

^b^These are error nodes; all errors are described in Multimedia Appendix 1.

**Table 3:** Top 10 errors by degree, closeness, betweenness, and PageRank in the error subgraph.^a^

Rank	Degree	Closeness	Betweenness	PageRank
1	Inadequate informed consent	Inadequate informed consent	Delay in treatment^b^	Inadequate informed consent
2	Unclear, ambiguous, illegible, or incomplete medical records	Unclear, ambiguous, illegible, or incomplete medical records	Lack of informed consent	Supervision or patient safety management
3	Supervision or patient safety management	Delay in treatment^b^	Failure to recognize complications^b^	Unclear, ambiguous, illegible, or incomplete medical records
4	Delay in treatment^b^	Failure to perform preoperative evaluation^b^	Failure to perform preoperative evaluation^b^	Failure to communicate with or instruct the patient or family
5	Failure to recognize complications^b^	Supervision or patient safety management	Unclear, ambiguous, illegible, or incomplete medical records	Lack of informed consent
6	Lack of informed consent	Failure to perform pretreatment evaluation^b^	Untimely patient rounds^b^	Emergency management
7	Failure to communicate with or instruct the patient or family	Failure to identify postoperative complications^b^	Failure to perform pretreatment evaluation^b^	Unsigned consent documentation
8	Failure to perform pretreatment evaluation^b^	Lack of informed consent	Delay in diagnosis^b^	Administrative management
9	Other surgery-related errors^b^	Delay in diagnosis^b^	Delay in surgery^b^	Other management-related errors
10	Other treatment-related errors^b^	Delay in surgery^b^	Other medicine-related errors^b^	Risk management

^a^All errors are described in Multimedia Appendix 1.

^b^Attributed to technical errors.

## Discussion

### Principal Findings

This study constructed a KG derived from medical malpractice litigation data to represent the MMNC. We found that the MMNC was a scale-free network instead of a random network. Scale-free networks representing the MMNC were high clustering, showed skewed degree distributions, and had hub nodes. The results of the hub nodes revealed that orthopedics, obstetrics and gynecology, and the emergency department were the 3 most frequent specialties that incurred medical malpractice; inadequate informed consent work constituted the most errors. Nontechnical errors (eg, inadequate informed consent) showed a higher centrality than technical errors.

Power laws are being discovered in a great number and with various phenomena; accordingly, some authors have described them as “more normal than ‘normal’” [54]. Power laws rarely emerge in systems completely dominated by a roll of the dice [55]. Thus, the power law that we observed with the MMNC signified that real networks are far from random. Plausible explanations for the nonrandom nature of the MMNC described in this study include the involvement of various human factors or errors. In the United States, the National Practitioner Data Bank classifies medical errors according to malpractice allegations, but subclassified terms are not further defined [56]. Numerous studies [57,58] have investigated the causal factors of medical malpractice by developing various human factor classification frameworks. Many countries have established adverse event reporting systems and classified those events—it is on such classification that the WHO-ICPS, referenced in this study, is based. However, those classification frameworks have not been widely used worldwide, and some frameworks have yet to be improved.

In complex theory, the widely accepted explanation for the existence of most (if not all) scale-free networks in the real world is growth and preferential attachment (ie, a particular growth process for such networks), as proposed by Barabási and Albert [35]. Thus, each network starts with a core node and grows by adding new nodes. There are connections among nodes—as more nodes become connected, the number of connections that result is greater. In the context of medical malpractice, the more hospitals or physicians with poor malpractice records, the greater the likelihood that they will become involved in future such cases. This is in harmony with the idea of the Pareto law or principle, which is also known as the 80/20 rule [59]. Accordingly, there has to be some order behind these complex systems [46,55]. The causes of the power law found in the MMNC need to be further studied.

The network analysis help identify hub nodes for interventions. The inevitability of the existence of hub nodes in scale-free networks presents an opportunity for prevention and control of medical malpractice. Consistent with the findings of recent research [2-4,13,14,19,20,23], we found that specialties such as orthopedics, obstetrics and gynecology, and the emergency department incur a disproportionately large share of litigation cases. The specific reasons are unknown; however, potential explanations are that such specialties admit higher-risk patients, operate in higher-risk environments, or are subject to the “bad apple effect” (ie, repeatedly provide substandard care) [60]. The hospitals included in this study are the top tertiary hospitals across China compared with other levels of medical institutions, which have better medical resources and treat more patients with intractable diseases. Some specific specialties of these hospitals are more likely to have a high incidence of medical malpractice. Obstetrics and gynecology involves the health of both newborns and *puerpera*, whereas orthopedic diseases have a more intuitive impact on limb function and daily work. Patients with orthopedic diseases tend to expect dramatic improvements in limb function following a major procedure, but unsatisfactory treatment results might occur. Emergency patients tend to have acute onset or severe illness, especially when there is no family member around to sign the informed consent, and the risk of medical malpractice in such cases could be higher. The “bad apple effect” could be explained by the anchoring effect; that is, because of the cognitive errors, medical staff might repeatedly provide substandard care with certain medical errors. The cognitive errors might have formed from previously acquired information or experience, and such errors are like an anchor sinking to the bottom of the sea, holding medical staff’s thoughts in place. In fact, it is what we often refer to as a “preconceived” notion.

Compared with technical errors, nontechnical errors had greater centrality in this study. However, descriptive studies in this field [13,14] show that technical errors occur more frequently. Our findings suggest that it may be effective to improve nontechnical skills to reduce accidents [61]. Our findings demonstrated that one of the most prominent nontechnical errors involved inadequate informed consent. Informed consent has always been one of the most common medical errors in China. In total, 2 Chinese studies [62,63] found that 23% to 43% of medical lawsuits involved incomplete consent notification for patients. Owing to the information asymmetry between physicians and patients, coupled with the tense relationship between physicians and patients in China [18], patients’ doubts will trigger medical malpractice once medical staff are insufficient in risk notification. In addition, errors related to medical records were particularly prominent among nontechnical errors. A plausible explanation is that medical records are the main evidence in the mediation of medical malpractice in China, and irregular writing will directly affect the judgment of medical litigation [14].

We found that the dominant factors in technical errors were inadequate attention and delays, including treatment delay, failure to recognize complications, and delays in surgery and diagnosis. Unlike in the United States, where diagnostic errors are the most common cause of malpractice claims [64,65], treatment and surgical errors are more frequent in China. The difference may be due to the fact that the medical system in the United States may be relatively fragmented (eg, the diagnosis and treatment of the same patient may be divided into different institutions, resulting in medical staff often diagnosing based on more fragmented information). Diagnostic errors may be ignored in China as medication and surgical errors are more easily observed during medical treatment. There is still considerable room in China for enhancing the quality of health care and patient safety management. There are variations in trends of technical errors in different specialties in China; however, there may be common interventions for nontechnical errors. For example, shared decision-making approaches can be and have been applied to all specialties; this helps protect physicians from malpractice claims and ensures that patients are better informed [66].

This study has found a number of hub nodes in the MMNC, including technical and nontechnical errors, which could be helpful for preventive education for medical malpractice. Nontechnical errors occupy an important position in the MMNC, reflecting the lack of awareness of error prevention in medical institutions and their medical staff. Compared with technical errors, nontechnical errors related to informed consent notification, physician-patient communication skills, and medical record writing could be relatively easily avoided by strengthening related training. However, the education of medical students in China places the most emphasis on clinical skills and scientific research, and training to avoid medical errors, especially nontechnical errors, is very limited. We believe that medical education and training should be strengthened to constantly improve clinical performance and the awareness of nontechnical errors among medical students and staff.

Network analysis provides a useful tool for analyzing medical malpractice. It does not require a complete map of medical malpractice, only measuring the degree distribution by analyzing a representative subset of the complete network [55]; we do so in this study. It is impossible to obtain medical malpractice data without omissions and build a complete malpractice network. Fortunately, a complete map of medical malpractice is not necessary to determine whether it is scale-free or random [55]. Another problem is identifying the hubs—doubtlessly, many hubs may have gone undiscovered in this study, and we may have included a few nonhubs. Decades of research have produced numerous graph methods for identifying hubs. Such methods may be imperfect, but they are still useful—it is possible to identify the hubs with a certain probability. Dezső and Barabási [10] demonstrated that any policy that displayed bias toward more connected nodes—even a small bias—restored the finite epidemic threshold. In the context of malpractice, it may not be possible to find all the hubs; however, by attempting to do so, the spread of medical malpractice can be limited. Network analysis is an emerging research field that has grown with the development of network theory and computer technology. In the real world, there are many fields that can be abstracted into complex networks. Physicists have found that power laws frequently signal a transition from disorder to order—such a distribution pattern is observed in most self-organized complex systems in nature, technology, and society [46,55]. Many people feel that they do not live in a random world—there have to be certain key organizational principles behind complex systems. Finding the rules hidden behind the structure in the MMNC is the next future direction.

### Limitations

This study had several limitations. First, medical malpractice litigation cases presumably represent the tip of the iceberg with medical errors, in which patients receive poor-quality health care [67]. Second, we assumed that the Chinese judiciary system is fair, independent, and strong; however, there are several deficiencies or flaws in medical malpractice law in China. Finally, simplified network models cannot explain everything regarding their real-world counterparts. With the MMNC, we assumed that all the nodes were identical except for their degree and that all links were of the same type and had the same strength; however, that is not the case in real-world networks.

### Conclusions

This study constructed a KG derived from medical malpractice litigation data to represent the MMNC. We demonstrated that it was a scale-free network, not a random network, and showed that the occurrence of medical malpractice was traceable. The MMNC was in transition from chaos to order, reflecting from the results of the hub nodes that there were several key specialties and errors. Faced with limited resources, it is necessary to make specific interventions for key specialties and errors as well as pay greater attention to nontechnical errors; doing so could effectively control medical risks.

## Appendix

Multimedia Appendix 1 Description of all medical errors.

Multimedia Appendix 2 Distribution of malpractice claims characteristics by data sample.

## Abbreviations

KG: knowledge graph
MMNC: medical malpractice network of China
WHO-ICPS: International Classification of Patient Safety from the World Health Organization

## Data Availability

Deidentified individual participant data that underlie the results reported in this paper (tables, figures, and multimedia appendices) can be made available to researchers who apply for proposed use after approval by the corresponding author of this study. A signed data access agreement is also needed for data requesters.
