# Correction: The Application of Graph Theoretical Analysis to Complex Networks in Medical Malpractice in China: Qualitative Study

**DOI:** 10.2196/44374

**Published:** 2022-12-07

**Authors:** Shengjie Dong, Chenshu Shi, Wu Zeng, Zhiying Jia, Minye Dong, Yuyin Xiao, Guohong Li

**Affiliations:** 1 School of Public Health, Shanghai Jiao Tong University Shanghai China; 2 Shuguang Hospital Affiliated to Shanghai University of Traditional Chinese Medicine Shanghai China; 3 Center for Health Technology Assessment, China Hospital Development Institute, Shanghai Jiao Tong University Shanghai China; 4 Department of International Health, Georgetown University Washington, DC United States; 5 Renji Hospital, School of Medicine, Shanghai Jiao Tong University Shanghai China; 6 China Hospital Development Institute Shanghai Jiao Tong University Shanghai China

In “The Application of Graph Theoretical Analysis to Complex Networks in Medical Malpractice in China: Qualitative Study” (JMIR Med Inform 2022;10(11):e35709) the authors noted one error:

The phone number of the corresponding author Guohong Li was corrected to 86 21 63846590.

The correction will appear in the online version of the paper on the JMIR Publications website on December 7, 2022 together with the publication of this correction notice. Because this was made after submission to PubMed, PubMed Central, and other full-text repositories, the corrected article has also been resubmitted to those repositories.

