# Supplementary material for: The Application of Graph Theoretical Analysis to Complex Networks in Medical Malpractice in China: Qualitative Study
Source: JMIR Med Inform. 2022 Nov 3;10(11):e35709. doi: 10.2196/35709 (PMC9673000; doi:10.2196/35709)
Supplement: Multimedia Appendix 1 [file medinform_v10i11e35709_app1.docx]

**Multimedia Appendix 1**

**Description of all medical errors**

| Type | Group | Error |
| --- | --- | --- |
| Technical Error | Diagnosis Related | Missed Diagnosis |
|  | Diagnosis Related | Delay in Diagnosis |
|  | Diagnosis Related | Failure to Diagnose |
|  | Diagnosis Related | Other Diagnosis Related |
|  | Anesthesia Related | Wrong Patient in Anesthesia |
|  | Anesthesia Related | Wrong Anaesthetic |
|  | Anesthesia Related | Wrong Dosee/Strength in Anesthesia |
|  | Anesthesia Related | Wrong Route in Anesthesia |
|  | Anesthesia Related | Improper Choice of Anesthetic Method |
|  | Anesthesia Related | Improper Order in Anesthesia |
|  | Anesthesia Related | Other Anesthesia Related |
|  | Surgery Related | Failure to Perform Pre-operative Evaluation |
|  | Surgery Related | Contraindication to Surgery |
|  | Surgery Related | Delay in Surgery |
|  | Surgery Related | Wrong Patient in Surgery |
|  | Surgery Related | Wrong Body Part/Side/Site in Surgery |
|  | Surgery Related | Improper Choice of Operation Methods |
|  | Surgery Related | Improper Order in Surgery |
|  | Surgery Related | Omitted Operation in Surgery |
|  | Surgery Related | Redundant Operation in Surgery |
|  | Surgery Related | Insufficient Sterilization in Surgery |
|  | Surgery Related | Intubation Problem |
|  | Surgery Related | Retained Foreign Body |
|  | Surgery Related | Unnecessary Procedure |
|  | Surgery Related | Unqualified Practitioners in Surgery |
|  | Surgery Related | Failure to Recognize Complications |
|  | Surgery Related | Other Surgery Related |
|  | Obstetrics Related | Improper Choice of Delivery Method |
|  | Obstetrics Related | Failure to Identify Fetal Distress |
|  | Obstetrics Related | Failure to Treat Fetal Distress |
|  | Obstetrics Related | Delay in Treatment of Identified Fetal Distress |
|  | Obstetrics Related | Other Obstetrics Related |
|  | Treatment Related | Failure to Perform Pre-treatment Evaluation |
|  | Treatment Related | Contraindication to Treatment |
|  | Treatment Related | Delay in Treatment |
|  | Treatment Related | Wrong Patient in Treatment |
|  | Treatment Related | Improper Choice ofTreatment Pragram |
|  | Treatment Related | Wrong Body Part/Side/Site in Treatment |
|  | Treatment Related | Improper Order in Treatment |
|  | Treatment Related | Omitted Operation in Treatment |
|  | Treatment Related | Insufficient Sterilization in Treatment |
|  | Treatment Related | Unnecessary Treatment |
|  | Treatment Related | Unqualified Practitioners in Treatment |
|  | Treatment Related | Other Treatment Related |
|  | Medication/IV Fluids Related | Prescribing to Drug/IV Fluids |
|  | Medication/IV Fluids Related | Wrong Drug |
|  | Medication/IV Fluids Related | Wrong Patient to Drug/IV Fluids |
|  | Medication/IV Fluids Related | Wrong Route to Drug/IV Fluids |
|  | Medication/IV Fluids Related | Contraindication to Medication/IV Fluids |
|  | Medication/IV Fluids Related | Wrong Formulation or Presentation to Drug/IV Fluids |
|  | Medication/IV Fluids Related | Wrong Frequency to Drug/IV Fluids |
|  | Medication/IV Fluids Related | Wrong Dose/Strength to Drug/IV Fluids |
|  | Medication/IV Fluids Related | Omitted Medicine or Dose |
|  | Medication/IV Fluids Related | Unnecessary Drugs |
|  | Medication/IV Fluids Related | Adverse Drug Reaction |
|  | Medication/IV Fluids Related | Wrong Quantity to Drug/IV Fluids |
|  | Medication/IV Fluids Related | Wrong Storage to Drug/IV Fluids |
|  | Medication/IV Fluids Related | Expired Drug |
|  | Medication/IV Fluids Related | Complementary/Alternative Drug |
|  | Medication/IV Fluids Related | Illegal Drug |
|  | Medication/IV Fluids Related | Wrong Dispensing Label/Instruction to Drug/IV Fluids |
|  | Medication/IV Fluids Related | Other Medication/IV Fluids Related |
|  | Blood/Blood Products Related | Prescribing to Blood/Blood Products |
|  | Blood/Blood Products Related | Wrong Patient to Blood/Blood Products |
|  | Blood/Blood Products Related | Wrong Blood Type |
|  | Blood/Blood Products Related | Wrong Blood Product |
|  | Blood/Blood Products Related | Contraindication to Blood Transfusion |
|  | Blood/Blood Products Related | Wrong Frequency to Blood/Blood Products |
|  | Blood/Blood Products Related | Wrong Dose/Strength to Blood/Blood Products |
|  | Blood/Blood Products Related | Unnecessary Blood Transfusion |
|  | Blood/Blood Products Related | Adverse Effect to Blood Transfusion |
|  | Blood/Blood Products Related | Wrong Quantity to Blood/Blood Products |
|  | Blood/Blood Products Related | Wrong Storage to Blood/Blood Products |
|  | Blood/Blood Products Related | Expired Blood/Blood Products |
|  | Blood/Blood Products Related | Wrong Dispensing Label/Instruction to Blood/Blood Products |
|  | Blood/Blood Products Related | Other Blood/Blood Products Related |
|  | Medical Device/Equipment Related | Wrong Quality to Medical Device/Equipmen |
|  | Medical Device/Equipment Related | Inappropriate for Task |
|  | Medical Device/Equipment Related | Unclean/Unsterile |
|  | Medical Device/Equipment Related | Failure/Malfunction |
|  | Medical Device/Equipment Related | Dislodgement/Misconnection/Removal |
|  | Medical Device/Equipment Related | User Error |
|  | Medical Device/Equipment Related | Irregular Inspection/Maintenance |
|  | Medical Device/Equipment Related | Illegal Device/Equipment |
|  | Medical Device/Equipment Related | Other Medical Device/Equipment Related |
|  | Nursing Related | Non-compliance |
|  | Nursing Related | Omitted Skin Test |
|  | Nursing Related | Failure to Deliver Therapeutic Diets |
|  | Nursing Related | Failure to Keep Patient Specimens |
|  | Nursing Related | Inadequate Aseptic Operation |
|  | Nursing Related | Unqualified Nurse |
|  | Nursing Related | Untimely Patient Rounds |
|  | Nursing Related | Pressure Ulcer |
|  | Nursing Related | Drainage Tube/ Intubation/ Needle Prolapse |
|  | Nursing Related | Nursing Transfer Problem |
|  | Nursing Related | Other Nursing Related |
| Non-technical Error | Medical Record Related | Medical Records Missing or Unavailable |
|  | Medical Record Related | Medical Records for Wrong Patient or Wrong Medical Records |
|  | Medical Record Related | Unclear/Ambiguous/Illegible/ Incomplete Medical Record |
|  | Medical Record Related | Altered or Misplaced Records |
|  | Medical Record Related | Prematurely Destroyed Records |
|  | Medical Record Related | Unqualified Recorder |
|  | Medical Record Related | Other Medical Record Related |
|  | Ethics Related | Lack of Informed Consent |
|  | Ethics Related | Unsigned Consent Documetation |
|  | Ethics Related | Inadequate Informed Consent |
|  | Ethics Related | Patient/Family Misunderstanding |
|  | Ethics Related | Wrong Patient/Family |
|  | Ethics Related | Unqualified Informer |
|  | Ethics Related | Breach of Patient Confidentiality |
|  | Ethics Related | Other Ethics Related |
|  | Commucation Related | Communication Problem Between Practitioners |
|  | Commucation Related | Failure to Instruct or Communicate with Patient or Family |
|  | Commucation Related | Other Commucation Related |
|  | Management Related | Claims/Risk Management |
|  | Management Related | Emergency Management |
|  | Management Related | Administrative Management |
|  | Management Related | Information Transfer |
|  | Management Related | Supervision/Patient Safety Management |
|  | Management Related | Other Management Related |
|  | Other | Religious Issues |
|  | Other | Assault and Battery/Sexual Misconduct |
|  | Other | Practitioner with Communicable Disease |
|  | Other | False Imprisonment |
|  | Other | Not Otherwise Classified, Specify |
