# Supplementary material for: The Application of Graph Theoretical Analysis to Complex Networks in Medical Malpractice in China: Qualitative Study
Source: JMIR Med Inform. 2022 Nov 3;10(11):e35709. doi: 10.2196/35709 (PMC9673000; doi:10.2196/35709)
Supplement: Multimedia Appendix 2 [file medinform_v10i11e35709_app2.docx]

**Multimedia Appendix 2**

**Distribution of Malpractice Claims Characteristics by Data Sample**

| **Plaintiff type——no(%)** | | **Closure date——no(%)** | |
| --- | --- | --- | --- |
| Inpatient | 6155(93.1) | 2008-2013 | 398(6.0) |
| Outpatient | 447(6.8) | 2013 | 256(3.9) |
| Not clear | 8(0.1) | 2014 | 932(14.1) |
| **Plaintiff gender——no(%)** | | 2015 | 1025(15.5) |
| Male | 1849(28.0) | 2016 | 977(14.8) |
| Female | 1761(26.6) | 2017 | 1624 (24.5) |
| Not clear | 3000(45.4) | 2018 | 1402 (21.2) |
| **Medical specialty——no(%)** | | **Area——no(%)** | |
| Orthopaedics | 758(11.5) | East China | 2794(42.3) |
| Obstetrics & gynecology | 634(9.6) | North China | 1331(20.1) |
| Emergency department | 607(9.2) | Central China | 724(10.9) |
| Oncology | 534(8.1) | Northeast China | 659(10.0) |
| General surgery | 492(7.4) | South China | 427(6.5) |
| Oncology | 491(7.4) | Northwest China | 347(5.3) |
| Cardiology | 344(5.2) | Southwest China | 328(4.9) |
| Neurosurgery | 319(4.8) | **Outcome of litigation——no(%)^a^** | |
| Neurology | 292(4.4) | No payment | 1527(23.1) |
| Respiratory Medicine | 284(4.3) | Equitable payment | 262(4.0) |
| Other | 1855(28.1) | Fault payment | 4821(72.9) |
| **Severity of injury——no(%)** | | **Proportionate liability——%^b^** | |
| None | 1789(27.1) | Mean | 41 |
| Minor injury | 1455(22.0) | Median | 40 |
| Severe injury | 1093(16.5) | **Amount of compensation paid——$^c^** | |
| Death | 2217(33.5) | Mean | 3531.3 |
| Mental injury only | 56(8.4) | Median | 21875.0(625.0,35937.5) |

**^a^** In China, hospitals bear the compensation in two ways: “fault payment” and “equitable payment”. Fault payment means that the patients’ injury is attributed to negligence. Equitable payment means that although the hospital is not at fault (without negligence), it provides appropriate compensation for the injured patients for humanitarian reasons.

^b^ Proportionate liabilities were calculated on the basis of fault paid claims only (n=4821). In China, when an error is found in a malpractice claim, it does not mean that the hospital will take full responsibility over the compensation. In this instance, an expert appraisal group shall analyze the liability degree of the negligence related to the injury. Eventually, every claim was distributed with a specific responsibility ratio (ranges between 0% and 100%).

^c^ Values are given in 2018 dollars, discounting by consumer price index ($U.S.1=¥ 6.4). Compensation amounts were calculated on the basis of fault paid claims (n=4821).
